# EDIL3/Del-1-Dependent Induction of AMPKβ Phosphorylation Regulates the Progression of Mesenchymal Stem-like Triple-Negative Breast Cancer

**DOI:** 10.3390/ijms27062679

**Published:** 2026-03-15

**Authors:** Seol-Hwa Jeong, Soo Jung Lee, In Hee Lee, Jeeyeon Lee, Byeongju Kang, Joon Suk Moon, Ho Yong Park, Ji Young Park, Nora Jee Young Park, Eun Ae Kim, Jieun Kang, Yee Soo Chae

**Affiliations:** 1 Cell & Matrix Research Institute, Kyungpook National University, Daegu 41944, Republic of Korea; jseolh@gmail.com; 2 Department of Oncology/Hematology, Kyungpook National University Chilgok Hospital, Kyungpook National University School of Medicine, Daegu 41404, Republic of Korea; majestio@hanmail.net (S.J.L.); cakey83@hanmail.net (I.H.L.); 3 Department of Breast Surgery, Kyungpook National University Chilgok Hospital, Kyungpook National University School of Medicine, Daegu 41404, Republic of Korea; j.lee@knu.ac.kr (J.L.); libertas033@gmail.com (B.K.); joonsukm@gmail.com (J.S.M.); phy123@knu.ac.kr (H.Y.P.); 4 Department of Pathology, Kyungpook National University Chilgok Hospital, Kyungpook National University School of Medicine, Daegu 41404, Republic of Korea; jyparkmd@knu.ac.kr (J.Y.P.); pathpjy@naver.com (N.J.Y.P.); 5 Breast Cancer Precision Medicine Institute, Kyungpook National University Chilgok Hospital, Daegu 40405, Republic of Korea; llmllmllmllx@naver.com (E.A.K.); hermione-j@hanmail.net (J.K.)

**Keywords:** EDIL3, Del-1, AMP-activated protein kinase β subunit, triple-negative breast cancer

## Abstract

Triple-negative breast cancer (TNBC) lacks effective targeted therapies, and the mechanisms by which developmental endothelial locus-1 (EDIL3/Del-1) promotes TNBC remain incompletely defined. We profiled Del-1 and AMPK subunits in TNBC cell lines by RT-PCR and immunoblotting, performed functional assays in CRISPR/Cas9 Del-1 knockout and AMPKβ-manipulated cells, and evaluated AMPKβ in early-stage TNBC tumors using tissue microarrays (TMA) (immunohistochemistry; *n* = 100) and *AMPKβ2* mRNA quantification. Del-1 and AMPKβ were enriched in TNBC cells, most prominently in the mesenchymal stem-like subtype, whereas AMPKα levels were relatively stable. Increased Del-1 and activated AMPKβ enhanced proliferation and invasion, while Del-1 deletion reduced AMPKβ expression and suppressed tumor-promoting phenotypes. Mechanistically, Del-1 increased AMPKβ phosphorylation at serine 108, and a phospho-mimetic AMPKβ mutant further amplified oncogenic effects. In the pilot TMA study, high AMPKβ protein expression showed a trend toward poorer DFS in Kaplan–Meier analysis, while multivariate analysis identified high AMPKβ protein expression as an independent factor associated with poorer DFS in patients with early TNBC. These data support AMPKβ as a key mediator of Del-1-driven signaling and suggest AMPKβ could be a therapeutic target in aggressive TNBC subsets.

## 1. Introduction

Breast cancer is the most commonly diagnosed cancer worldwide and remains a leading cause of cancer-related mortality in women [1]. Its biological heterogeneity often results in relapses and incurable metastatic disease, which are associated with therapeutic resistance and poor prognosis. To improve prognostic evaluation and guide treatment decisions in this heterogeneous disease, multiple prognostic factors have been identified, including traditional indicators such as tumor stage and histological grade, as well as molecular subtypes and specific genetic alterations [2].

Triple-negative breast cancer (TNBC), lacking estrogen receptors (ER), progesterone receptors (PR), and HER2, represents 10–15% of breast cancer cases [3]. TNBC carries a higher risk of recurrence and metastasis to critical organs such as the brain and lungs, leading to poorer survival outcomes than those of luminal breast cancer subtypes [4,5,6]. Its high mortality is partly due to the absence of target receptors for endocrine or trastuzumab-based treatments [7]. While recent advances with PARP inhibitors and immune checkpoint agents have improved survival, significant unmet clinical needs remain.

Against this background, studies investigated the role of developmental endothelial locus-1 (Del-1), also known as EDIL3, as a potential molecular target and proposed that Del-1, present on circulating extracellular vesicles (exosomes) or within tumor tissue, could serve as an innovative diagnostic or prognostic marker for early breast cancer [8,9]. Although Del-1 has been implicated in tumor progression, its mechanistic role in TNBC, particularly in aggressive contexts, remains unclear.

AMP-activated protein kinase (AMPK) functions as a cellular energy sensor that is activated under conditions of energy depletion, hypoxia, or inhibition of the mitochondrial respiratory chain [10]. It consists of a catalytic α subunit (α1 and α2), a scaffolding β subunit (β1 and β2), and a regulatory γ subunit (γ1, γ2, and γ3) [11]. Although the AMPK complex typically has a 1:1:1 stoichiometric ratio, various subunit combinations exist, and some isoforms are frequently amplified in tumor cells [10,12,13]. While AMPK research mainly focuses on the catalytic α subunit, the role of the β subunits remains underexplored; these subunits bind glycogen particles and may contribute to oncogenic signaling pathways [14,15].

Genomic profiling reveals that *PRKAB2* (AMPKβ2), located on chromosome 1q21, is commonly amplified in 1–30% of primary breast tumors and in over 70% of recurring cases [16,17]. Moreover, the oncogene Chromodomain Helicase DNA-binding protein 1-Like (*CHD1L*) is often co-amplified with *PRKAB2*, resulting in overexpression linked to antiapoptotic and pro-metastatic traits [17,18]. Collectively, these findings indicate that *PRKAB2* amplification may contribute to, and potentially drive, more aggressive tumor behavior in breast cancer.

Altogether, although both Del-1 and AMPKβ have been implicated in breast cancer progression, it remains unclear whether these two molecules are functionally linked in TNBC and whether such an interaction contributes to progression-related phenotypes. Based on our preliminary RNA-sequencing data suggesting a potential association between *Del-1* and *AMPKβ*, this study was designed to investigate their relationship using in vitro assays, molecular biology approaches, and patient-derived data analyses. Through this approach, we sought to determine whether a Del-1–AMPKβ axis may contribute to breast cancer progression and thereby provide additional insight into the molecular basis of aggressive TNBC.

## 2. Results

### 2.1. Del-1 Knockout Suppresses Breast Cancer Cell Metastasis

**Figure 1:**
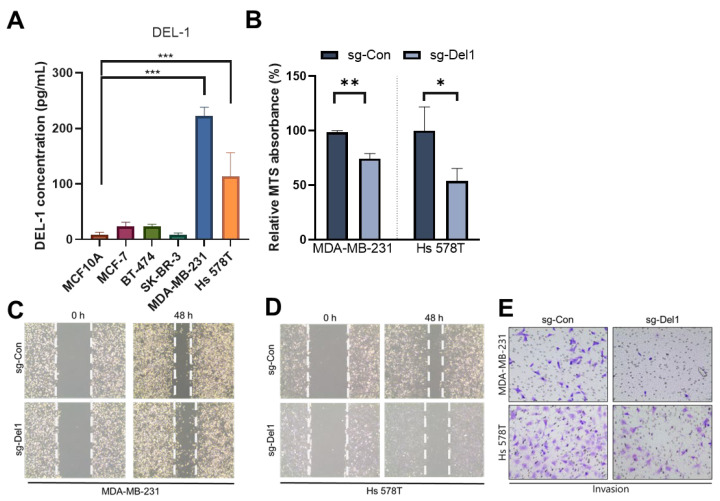
Depleted Del-1 suppresses breast cancer cell metastasis. (**A**) Del-1 protein levels were quantified using ELISA in the indicated cells. (**B**) MTS assays assessed cell viability in sg-Del1 MDA-MB-231 and Hs 578T cells after 24 h. (**C**,**D**) Migration assays evaluated the migratory capacity of MDA-MB-231 (**C**) and Hs 578T (**D**) cells with sg-Del1 compared to those of control cells. (**E**) Transwell invasion assays measured cellular invasiveness in sg-Del1 cells after 24 h. * *p* < 0.05, ** *p* < 0.01, and *** *p* < 0.001. Del-1, developmental endothelial locus-1; ELISA, enzyme-linked immunosorbent assay; sg-Del1, Del-1 knockout. *n* ≥ 5.

To investigate the role of Del-1 in breast cancer cells, we analyzed its expression using ELISA across four major subtypes: luminal A, luminal B, HER2-positive (HER2+), and TNBC. Del-1 was markedly upregulated in the TNBC cell lines MDA-MB-231 and Hs 578T (Figure 1A), both classified as the MSL subtype of TNBC, consistent with our previous findings [19].

To evaluate the role of Del-1 in the progression of MSL-type breast cancer, Del-1 in MDA-MB-231 and Hs 578T cells was disrupted. CRISPR-Cas9 sg-Del1 decreased viability in both cell lines (Figure 1B). We then performed wound-healing and transwell invasion assays, which revealed that Del-1 knockout significantly impaired the migratory and invasive capacities of MSL-type TNBC cells (Figure 1C–E).

### 2.2. Del-1 Silencing Downregulates AMPK Family Members in Breast Cancer Cells upon RNA Sequencing Analysis

**Figure 2:**
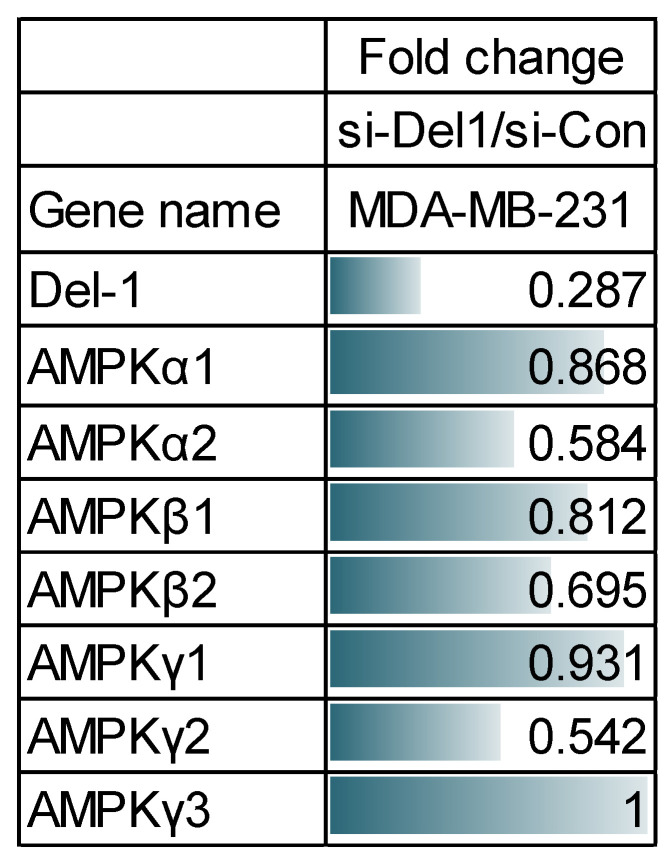
AMPK family members are downregulated in Del-1-silenced breast cancer cells. AMPK, AMP-activated protein kinase; Del-1, developmental endothelial locus-1.

Since Del-1 silencing blocked cancer cell invasion and viability in vitro, we investigated the molecular mechanisms through which Del-1 regulates breast cancer metastasis using RNA-sequencing. Analysis of the resulting data revealed that Del-1 silencing significantly reduced mRNA levels of AMPK families, key regulators of intracellular homeostasis (Figure 2).

### 2.3. Increased AMPKβ Expression Is Associated with Breast Cancer

**Figure 3:**
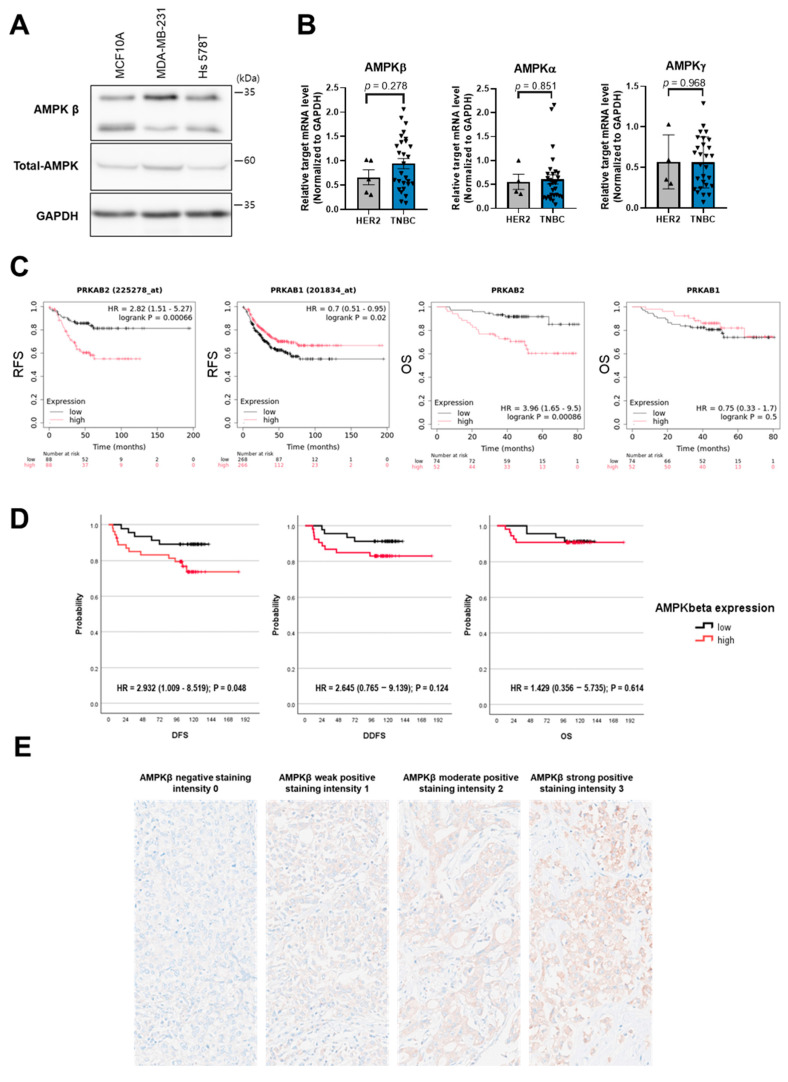
Increased AMPKβ is associated with TNBC (**A**) Western blot analysis of AMPK subunit protein levels in the indicated cells at 24 h. *n* ≥ 5. Data are representative of three independent experiments. (**B**) Real-time PCR analysis reveals the mRNA level of AMPK subunits AMPKβ, AMPKα, and γ (**C**) in human breast cancer tissues (HER2, *n* = 4; TNBC, *n* = 28). (**C**) Kaplan–Meier survival analysis for patients with breast cancer with high (red line) or low (black line) levels of AMPK β mRNA expression. (**D**) Survival curves based on AMPKβ expression in a cohort of 100 patients. (*n* = 100). Multivariate analysis is provided in Supplementary Table S2. (**E**) AMPKβ cytoplasmic staining was evaluated using a semi-quantitative approach: positivity was defined as ≥1% of tumor cells showing cytoplasmic staining, and staining intensity was graded as 0–3 (0, none; 1+, faint; 2+, intermediate; 3+, moderate-to-strong). GAPDH served as a loading control. DFS, disease-free survival; DDFS, distant disease-free survival; OS, overall survival; AMPK, AMP-activated protein kinase; HER2, human epidermal growth factor receptor 2; TNBC, triple-negative breast cancer; GAPDH, glyceraldehyde 3-phosphate dehydrogenase; PCR, polymerase chain reaction.

We then assessed the expression of AMPK family proteins in TNBC MSL cells with high Del-1 expression. In MDA-MB-231 and Hs 578T cell lines, AMPKβ protein levels were significantly upregulated (Figure 3A).

To determine whether this observation is reproducible in clinical samples, we examined the mRNA expression levels of AMPK subunits in the tissues of patients with breast cancer. Compared to other AMPK subunits, AMPKβ mRNA levels were higher in tissues from patients with TNBC (Figure 3B). We selected HER2+ patients’ samples as the control group, given the result that the Del-1 protein level was found to be the lowest in this subtype (Figure 1A). Consistent with these findings, analysis of the KM plotter database revealed that elevated AMPKβ2 mRNA expression (PRKAB2 225278_at) was significantly associated with advanced disease stages and poor relapse-free survival in patients with TNBC (Figure 3C), whereas AMPKβ1 mRNA expression showed no significant correlation with patient prognosis.

To validate the mRNA-based findings at the protein level, the expression of AMPKβ in human breast cancer tissues was examined by immunohistochemistry (IHC) using a tissue microarray (TMA). In a cohort of 100 patients with TNBC, high AMPKβ protein expression showed a trend toward poorer DFS in Kaplan–Meier survival analysis (*p* = 0.067; Figure 3D). In multivariable Cox regression analysis adjusted for age, tumor size, lymph node status, histologic grade, and Ki-67, high AMPKβ protein expression was significantly associated with poorer DFS (HR, 2.932; 95% CI, 1.009–8.519; *p* = 0.048), while only a nonsignificant trend toward poorer DDFS was observed (HR, 2.645; 95% CI, 0.765–9.139; *p* = 0.124) in patients with early TNBC (Supplementary Table S2). Detailed clinicopathological characteristics and additional survival information for this cohort are provided in the Supplement.

Collectively, these findings suggest that elevated expression of AMPKβ may be associated with increased Del-1 levels, an early marker of TNBC that is linked to poor clinical outcomes.

### 2.4. Pharmacological Activation of AMPKβ Promotes Proliferation and Invasion in MDA-MB-231 Cells

**Figure 4:**
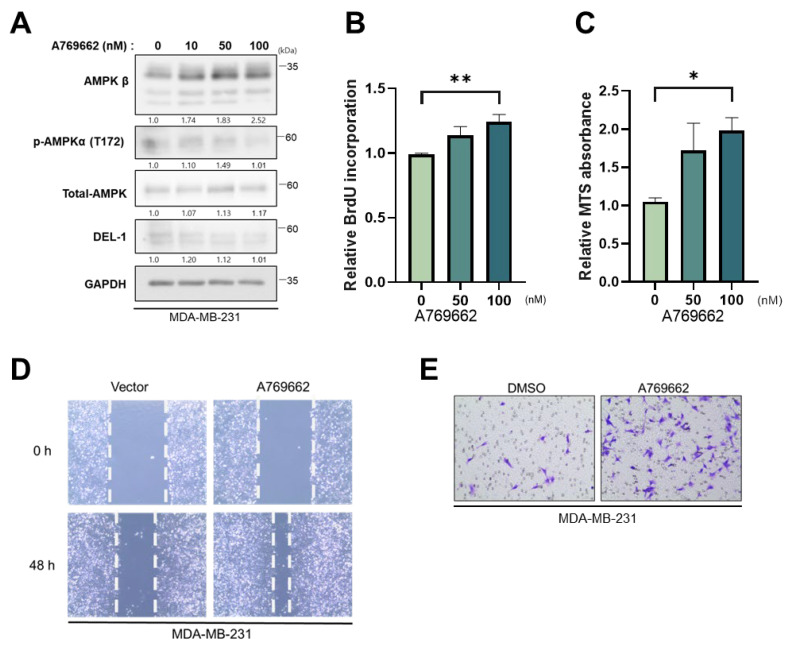
Pharmacological activation of AMPKβ promotes proliferation and invasion in MDA-MB-231 cells (**A**) Western blot analysis of protein expression in MDA-MB-231 cells treated with DMSO (0 nM) or the AMPKβ activator (10–100 nM) for 24 h. *n* ≥ 5 (**B**) BrdU proliferation assays reveal the effects of AMPKβ activator (100 nM) after 48 h. (**C**) MTS assays evaluated the proliferative effect of AMPKβ activator in MDA-MB-231 breast cancer cells. (**D**) Migration assays validated the migratory capacity of MDA-MB-231 cells exposed to the AMPKβ activator. (**E**) Transwell invasion assays measured invasiveness after 24 h of exposure to the AMPKβ activator (100 nM). GAPDH served as a loading control. Data are representative of three independent experiments. * *p* < 0.05 and ** *p* < 0.01. AMPK, AMP-activated protein kinase; DMSO, dimethyl sulfoxide; GAPDH, glyceraldehyde 3-phosphate dehydrogenase.

To investigate whether AMPKβ activation influences tumor progression, MDA-MB-231 cells were treated with A769662, a selective small-molecule activator of AMPKβ [13]. Stimulation with A769662 (100 nM, 24 h) resulted in a dose-dependent increase in AMPKβ expression, peaking at 100 nM (Figure 4A). While A769662 activates AMPKα via phosphorylation at T172 at certain concentrations [20], no detectable changes in AMPKα T172 phosphorylation were observed near 100 nM (Figure 4A), suggesting a selective effect on AMPKβ under these experimental conditions.

To further evaluate the biological significance of AMPKβ in breast cancer cell lines, its role in cell proliferation was investigated. MDA-MB-231 cells were treated with A769662 for 48 h, and proliferation was assessed using a BrdU incorporation assay. The results showed that A769662 treatment significantly enhanced the proliferative capacity of MDA-MB-231 cells compared to that of the controls, with the maximal effect observed at 100 nM (Figure 4B).

To examine whether AMPKβ activation affects cell viability in TNBC cells, MDA-MB-231 cells were incubated with A769662 at specific concentrations (50 nM and 100 nM) for 24 h, and their viability was assessed using an MTS assay. The viability of MDA-MB-231 was markedly increased following A769662 treatment (0–100 nM), with 100 nM producing a significant ~20% increase compared to that of the control group (Figure 4C). However, treatment with concentrations above 100 nM induced cytotoxicity in MDA-MB-231 cells.

Next, we examined the effect of AMPKβ activation on cell motility. A scratch wound-healing assay revealed that A769662 significantly increased the migratory ability of MDA-MB-231 cells after 48 h (Figure 4D). In addition to migration, the invasive capacity of the cells was elevated following 24 h of A769662 treatment, as assessed through a transwell invasion assay (Figure 4E).

Upregulation of AMPKβ did not significantly alter steady-state levels of Del-1 protein (Figure 4A), suggesting that AMPKβ acts downstream of Del-1 and that no autocrine feedback loop exists in the DEL-1–AMPKβ signaling axis.

Altogether, these findings indicate that AMPKβ is functionally active in TNBC cell lines and promotes tumor progression by enhancing proliferation, viability, migration, and invasion.

### 2.5. Del-1 Overexpression Enhances AMPKβ Expression and Drives Proliferation, Migration, and Invasion

**Figure 5:**
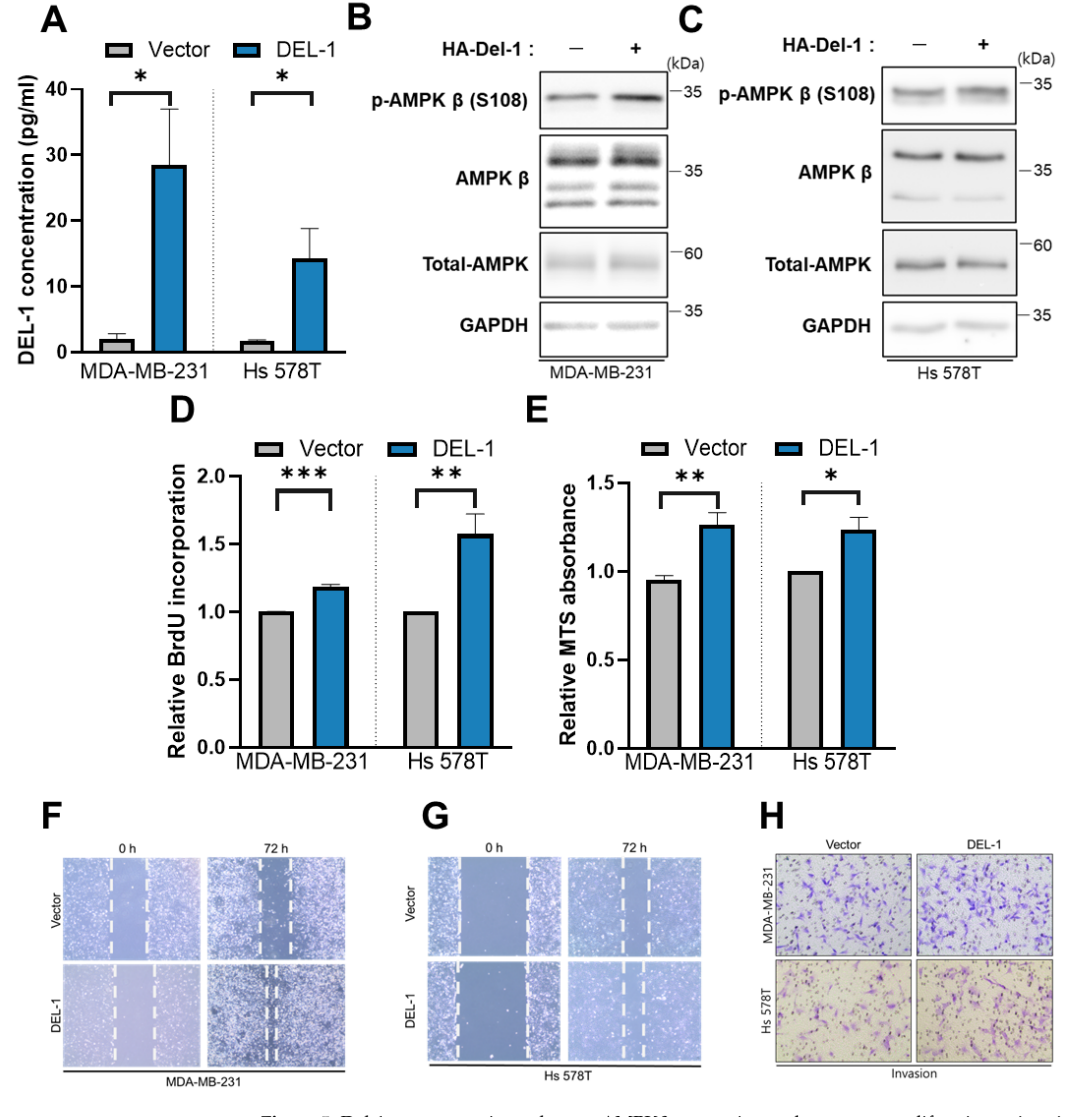
Del-1 overexpression enhances AMPKβ expression and promotes proliferation, migration, and invasion (**A**) ELISA measured Del-1 protein levels after overexpression in MDA-MB-231 and Hs 578T cells. (**B**,**C**) Western blot analysis reveals protein expression in MDA-MB-231 (**B**) and Hs 578T (**C**) cells following Del-1 overexpression. *n* ≥ 5 (**D**) BrdU proliferation assays demonstrate the effect of Del-1 overexpression compared with the control vector in MDA-MB-231 and Hs 578T cells after 48 h. (**E**) MTS assays evaluated the proliferative effect of Del-1 overexpression. (**F**,**G**) Wound-healing assays show the effects of Del-1 overexpression on cell migration for 72 h in MDA-MB-231 (**F**) and Hs 578T (**G**) cells. (**H**) Transwell matrix invasion assays illustrate the invasive capacity of MDA-MB-231 and Hs 578T cells after 48 h of Del-1 overexpression. GAPDH served as a loading control. Data are representative of 3 independent experiments. * *p* < 0.05, ** *p* < 0.01, and *** *p* < 0.001.

To further elucidate the mechanism underlying Del-1/AMPKβ signaling in cancer progression, we overexpressed Del-1 in MDA-MB-231 and Hs 578T cells. The AMPKβ protein levels were significantly higher in Del-1-overexpressing MDA-MB-231 cells than in the vector controls (Figure 5B), suggesting that AMPKβ may function as a downstream effector of the Del-1 signaling pathway. Furthermore, Del-1 overexpression markedly increased cell proliferation and viability (Figure 5D,E), supporting its role in MSL-type TNBC progression via AMPKβ activation.

While MDA-MB-231 and Hs 578T cells are both MSL TNBC cell lines with similar characteristics, such as stemness and high invasiveness [21,22], Del-1 overexpression affected AMPKβ expression differently in the two cell lines. In MDA-MB-231 cells, total AMPKβ and p-AMPKβ at serine 108 (S108) were upregulated. The S108 residue corresponds to a predicted phosphorylation site within the SXSXXD motif, as annotated in the PhosphoSitePlus database (6, 7). However, in Hs 578T cells, ectopic Del-1 expression increased p-AMPKβ (S108) levels without altering total AMPKβ expression. These findings indicate that AMPKβ activation—either through increased total expression or enhanced phosphorylation—may represent a critical event in Del-1–mediated signaling, although the underlying activation mechanisms appear to differ between cell lines.

### 2.6. Loss of Del-1 Reduces AMPKβ Levels

**Figure 6:**
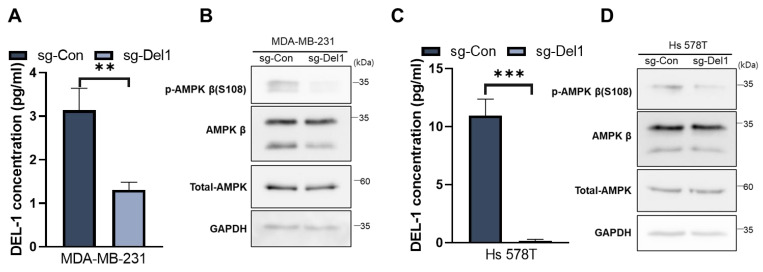
Del-1 knockdown reduces AMPKβ levels. (**A**,**C**) ELISA confirmed the Del-1 protein levels of CRISPR-Cas9-mediated Del1 knockout MDA-MB-231 (**A**) and Hs 578T cells (**C**). (**B**,**D**) Western blot analysis of related protein expression in MDA-MB-231 (**B**) and Hs 578T (**D**) cells with CRISPR-Cas9-mediated Del1 knockout. GAPDH served as a loading control. Data are representative of three independent experiments. ** *p* < 0.01 and *** *p* < 0.001. AMPK, AMP-activated protein kinase; Del-1, developmental endothelial locus-1; ELISA, enzyme-linked immunosorbent assay; GAPDH, glyceraldehyde 3-phosphate dehydrogenase. *n* ≥ 5.

To confirm the relationship between Del-1 and AMPKβ and to elucidate the influence of this signaling pathway on the metastatic potential of MSL-type TNBC cells, we employed the CRISPR-Cas9-mediated Del-1 knockout model. In Del-1 knockout MDA-MB-231 and Hs 578T cells, deletion of Del-1 downregulated total AMPKβ and p-AMPKβ (S108) (Figure 6C,D).

Overall, these findings support the conclusion that AMPKβ acts as a downstream effector of Del-1 and that the Del-1/AMPKβ axis plays a critical role in driving the progression of MSL TNBC.

### 2.7. Del-1-Mediated Phosphorylation of AMPKβ at S108 Is Crucial for TNBC Cell Metastasis

**Figure 7:**
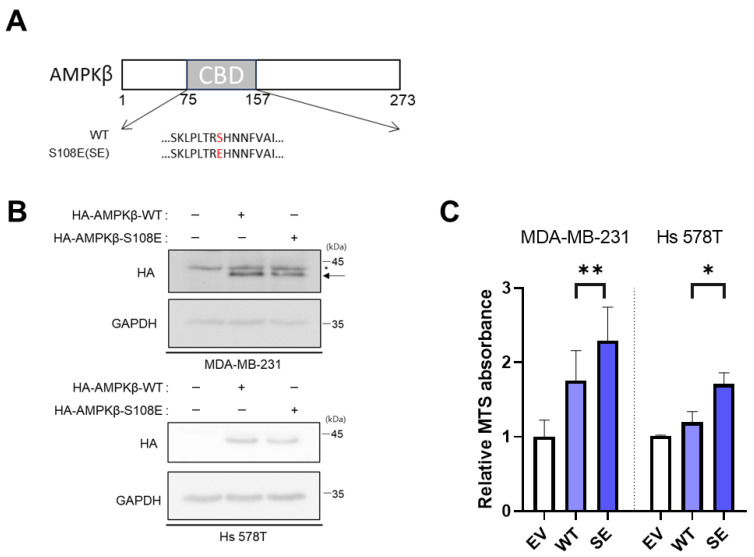
Del-1-mediated AMPK β phosphorylation at S108 is crucial for TNBC cell metastasis (**A**) Schematic diagram showing the genetic alteration of the phosphorylation site. (**B**) Western blot analysis of protein levels in MDA-MB-231 and Hs 578T cells expressing AMPKβ WT or its phosphomimetic form (SE). (**C**) MTS assays reveal the effects of AMPKβ WT and SE overexpression in MDA-MB-231 and Hs 578T cells. Data are representative of three independent experiments. GAPDH served as a loading control. * indicates a nonspecific band. The arrow represents both HA-AMPKβ WT and S1083E vectors. * *p* < 0.05 and ** *p* < 0.01. DEL-1, developmental endothelial locus-1; TNBC, triple-negative breast cancer; S108, serine 108 site; AMPK, AMP-activated protein kinase; GAPDH, glyceraldehyde 3-phosphate; WT, wild-type; SE, AMPKβ phosphomimetic form. *n* ≥ 5.

Since Del-1-mediated AMPKβ phosphorylation was confirmed in Figure 5, we hypothesized that p-AMPKβ acts as a trigger of MSL TNBC progression. To investigate the functional role of AMPKβ phosphorylation at S108, a phosphorylation-mimetic mutant was generated, in which S108 was substituted with glutamic acid (S108E) (Figure 7A). This substitution mimics constitutive phosphorylation at the AMPK S108 site. Transfection of AMPKβ S108E into MDA-MB-231 and Hs 578T cells resulted in a significant increase in cell viability compared to that of cells transfected with wild-type AMPKβ (Figure 7B,C). We also tested a phosphorylation-deficient mutant (S108A), but its effect on suppressing tumor progression was minimal. These findings highlight phosphorylation of AMPKβ at S108 as a critical regulatory mechanism that enhances cell viability and proliferation, thereby promoting TNBC progression.

## 3. Discussion

AMPK is a key regulator of energy balance at the cellular and systemic levels, coordinating nutrient availability with energy demand [23]. Transcriptome analysis revealed that suppression of Del-1 in MDA-MB-231 cells resulted in marked downregulation of AMPK family members. Additionally, high AMPKβ2 mRNA expression, as identified through KM plotter analysis, was associated with increased relapse risk and multivariate analysis identified high AMPKβ protein expression as an independent factor associated with poorer DFS in early TNBC in the clinical pilot cohort. Collectively, these findings prompted us to investigate the role of AMPKβ as a downstream effector of Del-1 in MSL TNBC subtypes.

Del-1 is significantly overexpressed in TNBC and regulates cancer progression through microRNAs, epithelial membrane protein 2, and maternal embryonic leucine zipper kinase (MELK) signaling pathways [19,24,25,26,27]. A study demonstrates that MELK is a druggable downstream effector of Del-1, particularly in MSL and basal-like TNBC subtypes [19]. The present study extends this signaling framework by identifying AMPKβ as a novel, subtype-specific target in Del-1–mediated oncogenic signaling.

Our results showed that Del-1 overexpression selectively upregulated AMPKβ phosphorylation at S108 in MSL TNBC cells (MDA-MB-231 and Hs 578T). This phosphorylation site is located within the glycogen-binding domain and mediates glycogen binding, a mechanism linked to enhanced metabolic plasticity in aggressive tumors [14,15]. Increased S108 phosphorylation further promoted cell proliferation, survival, and invasiveness, suggesting that the Del-1–p-AMPKβ(S108) axis functions as a key driver of MSL TNBC progression.

Notably, Del-1 and MSL share the characteristic of undergoing Epithelial–Mesenchymal Transition (EMT). The differential effect of Del-1 overexpression on MSL progression through AMPKβ activation, which is observed only in MSL, suggests that the EMT environment may play a modulatory role in the cell-type-specific regulation of the Del-1/AMPKβ signaling pathway. Therefore, further studies are essential to define the precise interplay between EMT and the Del-1/AMPKβ signaling cascade and to characterize the exact molecular mechanism responsible for this observed cell-type specificity. Future studies should further characterize the Del-1–dependent AMPKβ signaling axis, including whether AMPKβ modulates additional Del-1–associated molecular components.

To address the high mortality of TNBC, which is primarily driven by the absence of clear molecular targets, molecular and clinical subtyping is crucial for identifying novel therapeutic avenues and enhancing prognosis. The widely recognized studies by Lehmann classified TNBC into six subtypes: basal-like 1/2, mesenchymal, IM, MSL, and luminal androgen receptor [5,28]. Investigating subtype-specific vulnerabilities is essential for developing effective targeted therapies. The MSL subtype (11.5–23.9% of TNBC) is marked by EMT markers (e.g., FOXC1 and DCLK1) and an aggressive stem-like, invasive phenotype [5,29], contributing to its significantly higher incidence of metastasis [30,31,32]. This clinical behavior highlights the critical need for mechanistic insights into metastasis-specific signaling pathways in MSL TNBC, a gap addressed by our findings, indicating that Del-1–induced AMPKβ activation may represent such a mechanism.

Although AMPKα has been extensively studied as a tumor suppressor and a target of metformin-induced antiproliferative effects in ER+/PR+ breast cancer cells, its role appears attenuated in TNBC, as our study showed minimal changes in phosphorylated AMPKα (T172) [33,34]. The AMPKβ subunit, despite its well-established role in glycogen sensing and energy regulation, has been underexplored in breast cancer. Our data support a context-dependent, pro-tumorigenic association of AMPKβ in MSL-like TNBC models, in contrast to reports suggesting tumor-suppressive roles in other cancer types, such as colon or lung cancer [34,35].

While our findings strongly support the role of Del-1 as a central regulator of oncogenic kinases in TNBC, its potential as a direct therapeutic target remains limited. Del-1 is an extracellular matrix protein that circulates abundantly and plays essential roles in angiogenesis, immune modulation, and vascular homeostasis [36,37]. Directly targeting Del-1 may therefore pose a significant risk of systemic toxicity or immune-related adverse effects. Instead, targeting its downstream effectors, such as AMPKβ, whose expression and function are more restricted, may offer a more precise and clinically actionable approach.

The present study has several limitations. First, although the findings support an association between Del-1 and AMPKβ S108 phosphorylation, the precise upstream kinase and intermediary signaling pathway remain to be identified. Second, although PRKAB2/AMPKβ2 showed a stronger association than PRKAB1/AMPKβ1 in the KM plotter analysis, the patient IHC, CRISPR-Cas9 knock-out/gain-of-function, and pharmacologic experiments were not designed to distinguish isoform-specific contributions of AMPKβ1 and AMPKβ2. Importantly, because A769662 has a reported preference for β1-containing AMPK complexes, the pharmacologic findings should be interpreted as evidence of AMPK pathway involvement rather than as support for a PRKAB2/AMPKβ2-specific mechanism. Therefore, the current findings should be interpreted as supporting an AMPKβ-associated effect rather than a definitive isoform-specific mechanism. Finally, the EDIL3 knockout experiments were performed using a single sgRNA without complementary rescue experiments or validation using independent sgRNAs. Although a marked reduction in Del-1 concentration was confirmed by ELISA, potential off-target effects cannot be fully excluded.

In conclusion, our study identifies AMPKβ and its phosphorylation at S108 as critical downstream effectors of Del-1 signaling in the MSL subtype of TNBC. Given its subtype-specific activation pattern and strong functional effect on tumor progression, AMPKβ represents a promising therapeutic target in aggressive TNBC subsets. These findings expand the current understanding of Del-1–mediated signaling and provide a rationale for developing future therapeutics targeting AMPKβ in MSL TNBC.

## 4. Materials and Methods

### 4.1. Antibodies and Reagents

The following reagents and antibodies against the following proteins were used: A769662 (3336; Tocris, Bristol, United Kingdom); AMPKβ1/2 (4150), total AMPK (2532), and phosphorylated AMPKβ (p-AMPKβ; S108) (23021) from Cell Signaling Technology (Danvers, MA, USA); GAPDH (sc-47724) from Santa Cruz Biotechnology (Dallas, TX, USA); Del-1 (PA5-27994) from Thermo Fisher Scientific (Rockford, IL, USA); and HA (ab236632) from Abcam (Waltham, MA, USA).

### 4.2. Cell Culture

Breast cancer cell lines used included MCF10A (normal breast cell), MCF-7 (luminal A), BT-474 (luminal B), SK-BR-3 (HER2), MDA-MB-231 (TNBC), and Hs 578T (TNBC), all obtained from ATCC. Cells were cultured in a humidified incubator at 37 °C with 5% CO_2_ in Dulbecco’s Modified Eagle Medium (DMEM, high glucose; SH30243.01; Cytiva, Logan, UT, USA), supplemented with 10% fetal bovine serum (FBS; 16000044; Gibco, Grand Island, NE, USA). Cell lines were subcultured twice per week.

### 4.3. Establishment of CRISPR-Cas9-Engineered Knockout Cell Lines

Using the CRISPR/Cas9 system, Del-1 knockout cells (sg-Del-1) were generated by introducing a guide RNA targeting the human EDIL3 gene (NCBI Gene ID: 10085) into MDA-MB-231 and Hs 578T cells. A Cas9- and gRNA-encoded plasmid was transfected at a 1:5 ratio with Lipofectamine 3000 reagent (L3000015; Carlsbad, CA, USA), and cells were selected using puromycin after 24 h (2 μg/mL, p8833; Sigma Aldrich, St. Louis, MO, USA) according to the guidelines of the manufacturer. gRNA was designed and synthesized from Bioneer (Daejeon, Republic of Korea). EDIL3 gRNA target sequence (TGAAGCGCTCGGTAGCCGTCTGG).

### 4.4. Western Blotting

Cell lysates were prepared by scraping cells into radioimmunoprecipitation assay buffer (89901; Thermo Fisher Scientific, Rockford, IL, USA) containing 1 x protease and phosphatase inhibitor cocktail (78445; Thermo Fisher Scientific, Rockford, IL, USA) for 30 min with vortexing. After centrifugation, the protein concentrations were measured using a BCA protein assay reagent (23225; Thermo Fisher Scientific, Rockford, IL, USA). Typically, 20–30 μg of protein was loaded per lane and separated through SDS-polyacrylamide gel. Proteins were transferred onto nitrocellulose membranes (HATF085R; Millipore, Carrigtwohill, Ireland). Membranes were blocked with 5% nonfat dry milk and incubated overnight at 4 °C with primary antibodies. After washing, membranes were incubated for 1 h with horseradish peroxidase-conjugated secondary antibodies (7074; Cell Signaling Technology, Danvers, MA, USA). Protein bands were visualized using the ImageQuant LAS 500 system (Cytiva, Marlborough, MA, USA) with chemiluminescence detection. Band intensities were quantified using ImageJ software (NIH, version 1.54f) and normalized to GAPDH.

### 4.5. Matrigel Invasion Assay

The invasion chamber inserts were coated with Matrigel (356234; Corning, Tewksbury, MA, USA) for 4 h. MSL cells were trypsinized, resuspended in DMEM containing 1% FBS, and seeded into the upper chamber at a density of 2 × 10^4^ cells per well. The lower chamber was filled with control medium supplemented with 10% FBS. After 48 h, cells remaining on the upper surface of the inserts were removed with cotton swabs, and the membranes were stained with 1% crystal violet (V5265; Sigma-Aldrich, Darmstadt, Germany) and assessed under light microscopy (CKX53SF; Olympus, Tokyo, Japan).

### 4.6. BrdU Proliferation Assay

The proliferation assay was conducted using a BrdU Cell Proliferation ELISA kit (11647229001; Roche, Mannheim, Germany) according to the protocol of the manufacturer. The MSL cells were seeded in 96-well plates at a density of 2 × 10^3^ cells/well. After 48 h, the cells were labeled with BrdU and incubated with an anti-BrdU antibody. Following substrate addition, the colorimetric signal was assessed using a microplate reader (EPOCH; BioTek, Winooski, VT, USA).

### 4.7. MTS Assay

Cells were plated in 96-well plates at a density of 5 × 10^3^ cells per well. They were treated with CellTiter 96^®^ AQueous One Solution Cell Proliferation Assay (MTS; G3582; Promega, Madison, WI, USA) and incubated at 37 °C in a 5% CO_2_ incubator for 1 h. Absorbance was measured at 490 nm.

### 4.8. Migration Assay

Cells were cultured until they reached 90–100% confluence. Confluent monolayers were scratched using an SPL Scar Scratcher (201907; SPL Life Sciences, Pocheon, Republic of Korea) and rinsed twice with PBS. Images of the wound sites were captured at 0 and 48 h using a microscope (CKX53SF; Olympus, Tokyo, Japan) at fixed positions. Wound closure was quantified with ImageJ as the percentage reduction relative to baseline. Experiments were conducted in triplicate, and the assays were repeated at least three times.

### 4.9. RNA Sequencing

Del-1 was silenced using siRNA in MDA-MB-231 cells with pre-designed siRNA (4392420; Ambion, Austin, TX, USA), and RNA sequencing was conducted to evaluate transcriptomic alterations. RNA sequencing was performed as previously described [19].

Total RNA quality was assessed using the Agilent 2100 Bioanalyzer (Agilent Technologies Inc., Santa Clara, CA, USA) and quantified with the Nanodrop ND-2000 Spectrophotometer (Thermo Fisher Scientific Inc., Waltham, MA, USA). The isolation of mRNA was performed using the Poly(A) RNA Selection Kit (LEXOGEN, Inc., Vienna, Austria). Libraries were subsequently generated using the NEBNext^®^ Ultra™ II Directional RNA Library Prep Kit for Illumina^®^ (NEW ENGLAND BioLabs, Inc., Ipswich, MA, USA) following the manufacturer’s instructions. Library quality was verified using the Agilent 2100 Bioanalyzer (DNA High Sensitivity Kit), and final quantification was performed by RT-PCR. High-throughput sequencing was performed by assessing paired-end 100 bp reads on the Illumina^®^ NovaSeq 6000 platform (Illumina Inc., San Diego, CA, USA). Raw sequencing data quality control was conducted using FastQC v0.12.1. Adapter and low-quality sequences (<Q20) were removed using FASTX-Trimmer and BBMap. Cleaned reads were aligned to the reference genome using TopHat 2.1.2. Gene expression levels were quantified as FPKM (Fragments Per Kilobase per Million mapped reads) values using Cufflinks 2.2.1. The FPKM values were then subjected to Quantile normalization using custom scripts within the R environment.

### 4.10. RT-PCR

Total RNA was extracted from patient tissues with RNeasy Lipid Tissue Mini Kit (74804; QIAGEN, Hilden, Germany) and reverse-transcribed into cDNA. Quantitative real-time PCR was performed using the StepOnePlus system (Thermo Fisher Scientific, Singapore) and TaqMan Gene Expression Master Mix (3109063; Thermo Fisher Scientific, Vilnius, Lithuania). GAPDH was used as a reference gene. The primers were as follows: PRKAA1 (Assay ID: Hs01562315_m1), PRKAB1 (Assay ID: Hs00272166_m1), and PRKAG1 (Assay ID: Hs01091629_g1), all from Thermo Fisher Scientific.

### 4.11. Immunohistochemistry

To construct the TNBC TMA, 2 mm-diameter cores were extracted from formalin-fixed, paraffin-embedded tumor tissue blocks of 110 patients with early TNBC who underwent curative surgery. Representative tumor regions were selected in triplicate based on a review of the original H&E-stained slides by two pathologists.

AMPKβ expression was assessed using an automated BenchMark XT autostainer (Ventana Medical Systems, Tucson, AZ, USA) for IHC. TMA blocks were sectioned at 4 μm and mounted on coated glass slides, then heated at 60 °C for 2 h. Deparaffinization was performed with an EZ Prep solution (Ventana Medical Systems), followed by heat-induced antigen retrieval with CC1 solution from the same supplier. A 1:100 dilution of anti-AMPKβ antibody (Cell Signaling Technology, Danvers, MA, USA) was incubated for 1 h. Visualization was performed using a universal DAB detection kit (Ventana Medical Systems), with hematoxylin used for nuclear counterstaining. Negative controls were prepared by replacing the primary antibody with phosphate-buffered saline.

The immunohistochemical expression of AMPKβ was semi-quantitatively evaluated by assessing both the staining intensity and the proportion of positive tumor cells. Cytoplasmic staining was considered the primary localization for AMPKβ expression. A sample was defined as positive for AMPKβ when at least 1% of the tumor cells exhibited discernible cytoplasmic immunoreactivity. The staining intensity was further classified into a 4-tier scoring system. Two pathologists, blinded to clinicopathological data, independently assessed and assigned scores for AMPKβ immunoreactivity on the TMA.

### 4.12. Statistical Analysis

All values are presented as mean ± standard error of the mean (SEM). Statistical significance was validated using one-way analysis of variance (ANOVA) after Tukey’s post hoc test was used for multiple comparisons in GraphPad Prism version 8.0 (GraphPad Software, Boston, MA, USA). Survival analyses of patients with TNBC were conducted using SPSS version 25.0 (IBM Corp., Armonk, NY, USA), and *p*-values were calculated using Kaplan–Meier and Cox regression methods.

## Data Availability

The RNA-seq data generated and analyzed in this study are not publicly available at this time because they are part of an ongoing follow-up study with expanded analyses, but are available from the corresponding author upon reasonable request.

## Supplementary Materials

The following supporting information can be downloaded at: https://www.mdpi.com/article/10.3390/ijms27062679/s1.

## Abbreviations

The following abbreviations are used in this manuscript:

AMPK	AMP-activated protein kinase
sg-Del1	CRISPR-Cas9 Del-1 knockout
Del-1	developmental endothelial locus-1
HER2	human epidermal growth factor receptor 2
HER2+	HER2 positive
EMT	Epithelial–Mesenchymal Transition
ER	estrogen receptor
IHC	immunohistochemistry
IM	immunomodulatory
MELK	maternal embryonic leucine zipper kinase
MSL	mesenchymal stem-like
PR	progesterone receptor
TMA	Tissue microarray
TNBC	Triple-negative breast cancer
p-AMPKβ	phosphorylated AMPKβ
S108	serine 108 site
