# Supplementary material for: EDIL3/Del-1-Dependent Induction of AMPKβ Phosphorylation Regulates the Progression of Mesenchymal Stem-like Triple-Negative Breast Cancer
Source: Int J Mol Sci. 2026 Mar 15;27(6):2679. doi: 10.3390/ijms27062679 (PMC13026944; doi:10.3390/ijms27062679)

## Slide 1
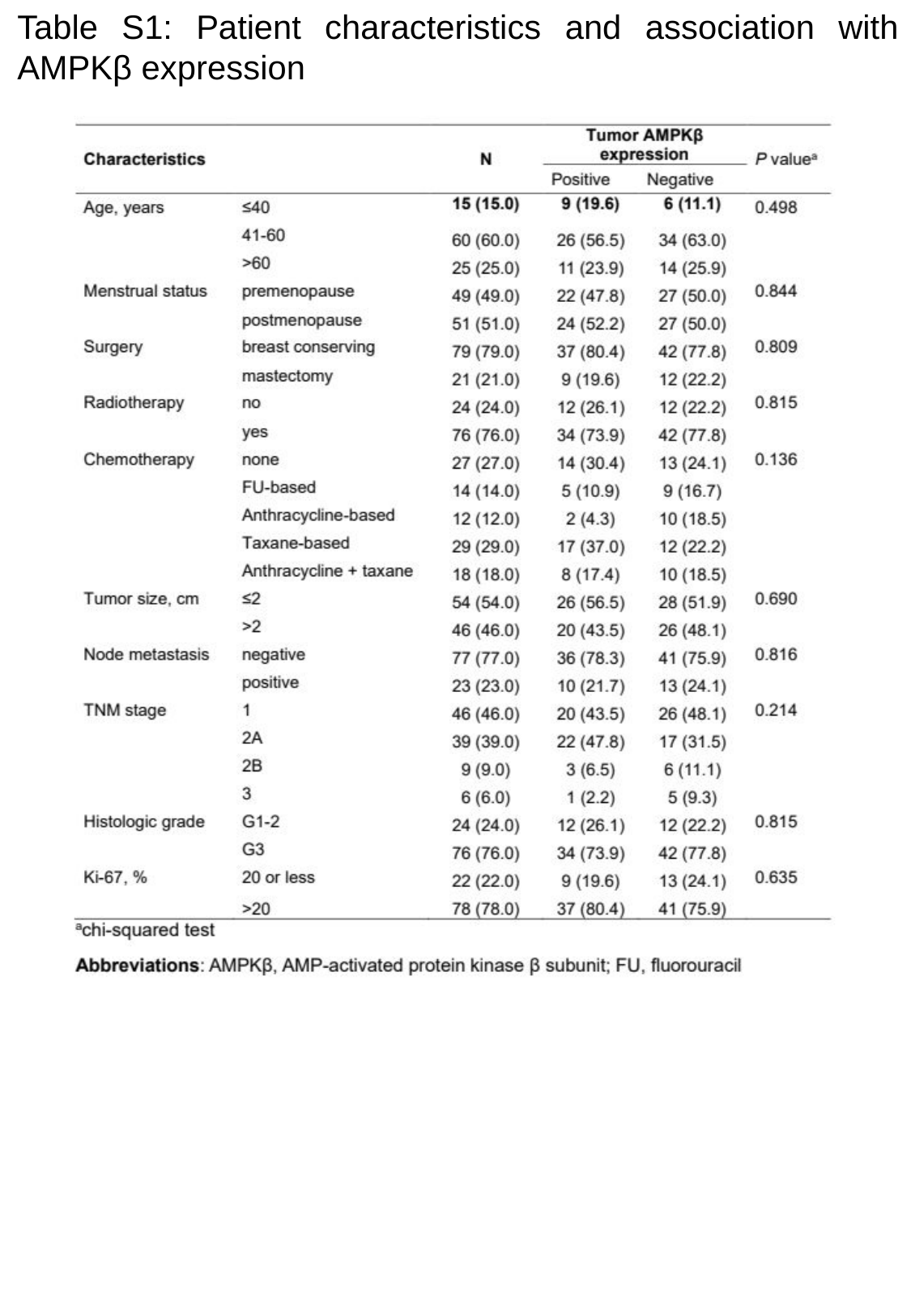

Table S1: Patient characteristics and association with AMPKβ expression

## Slide 2
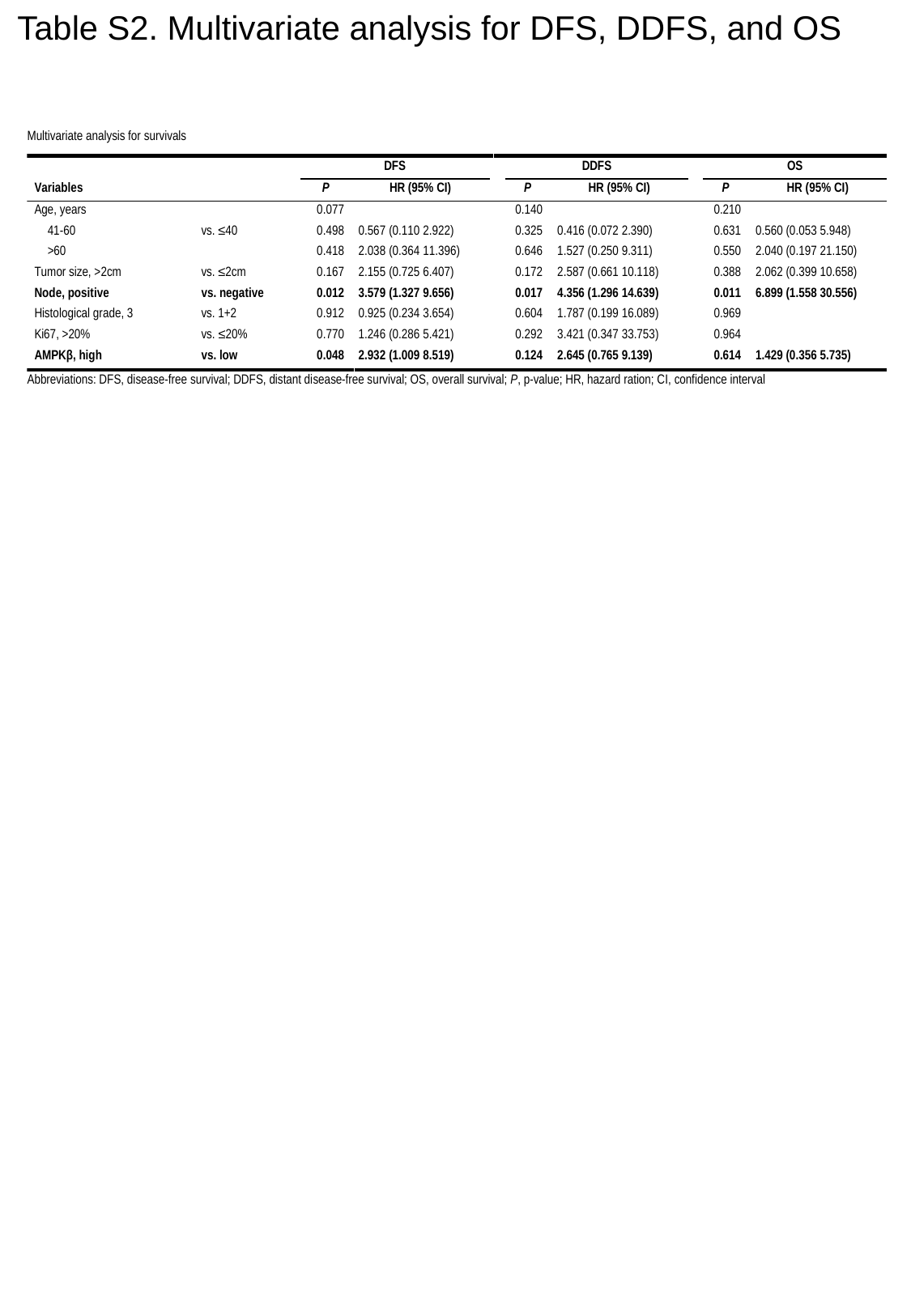

Table S2. Multivariate analysis for DFS, DDFS, and OS

## Slide 3
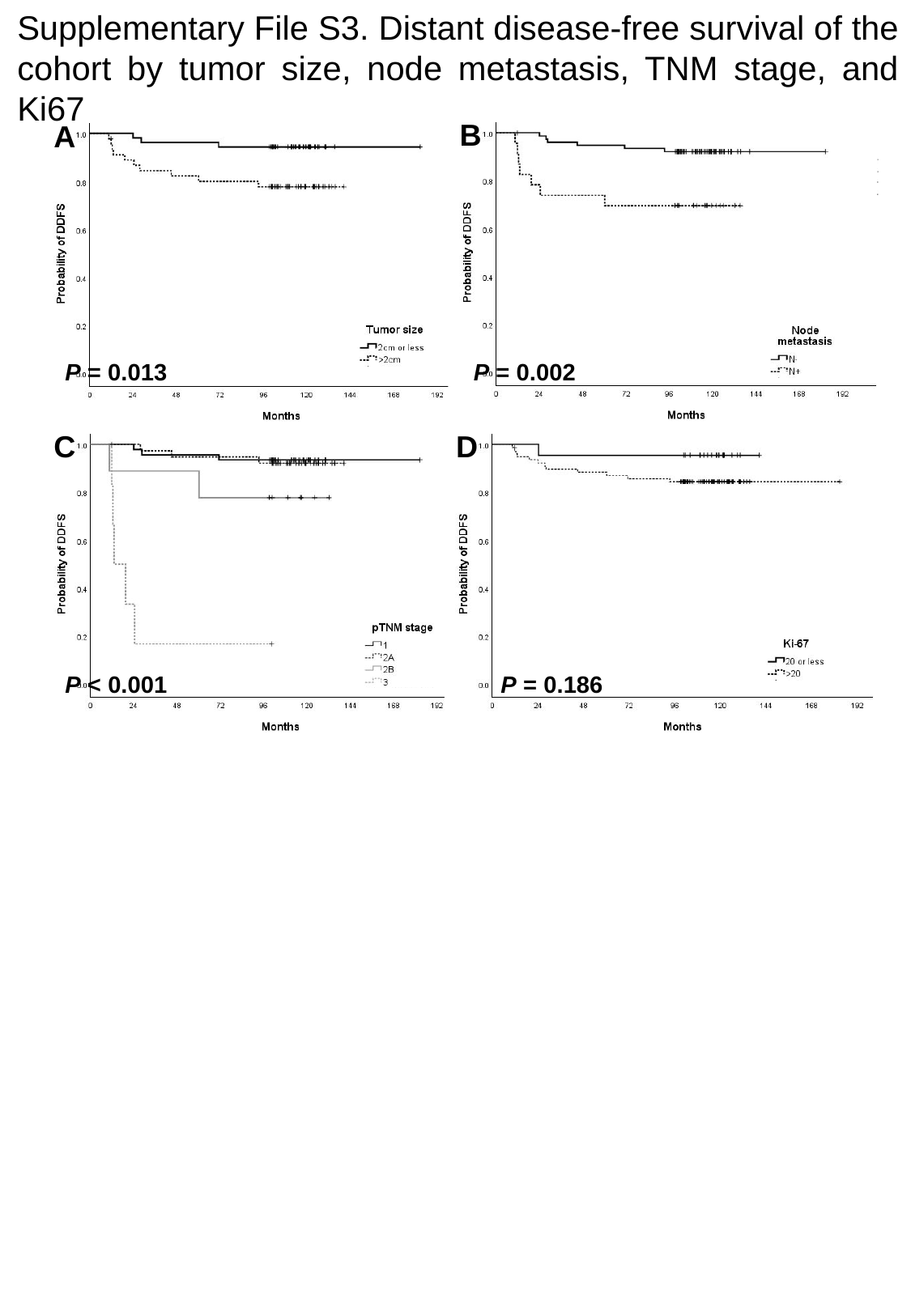

Supplementary File S3. Distant disease-free survival of the cohort by tumor size, node metastasis, TNM stage, and Ki67
B
P = 0.002
A
P = 0.013
C
P < 0.001
D
P = 0.186

## Slide 4
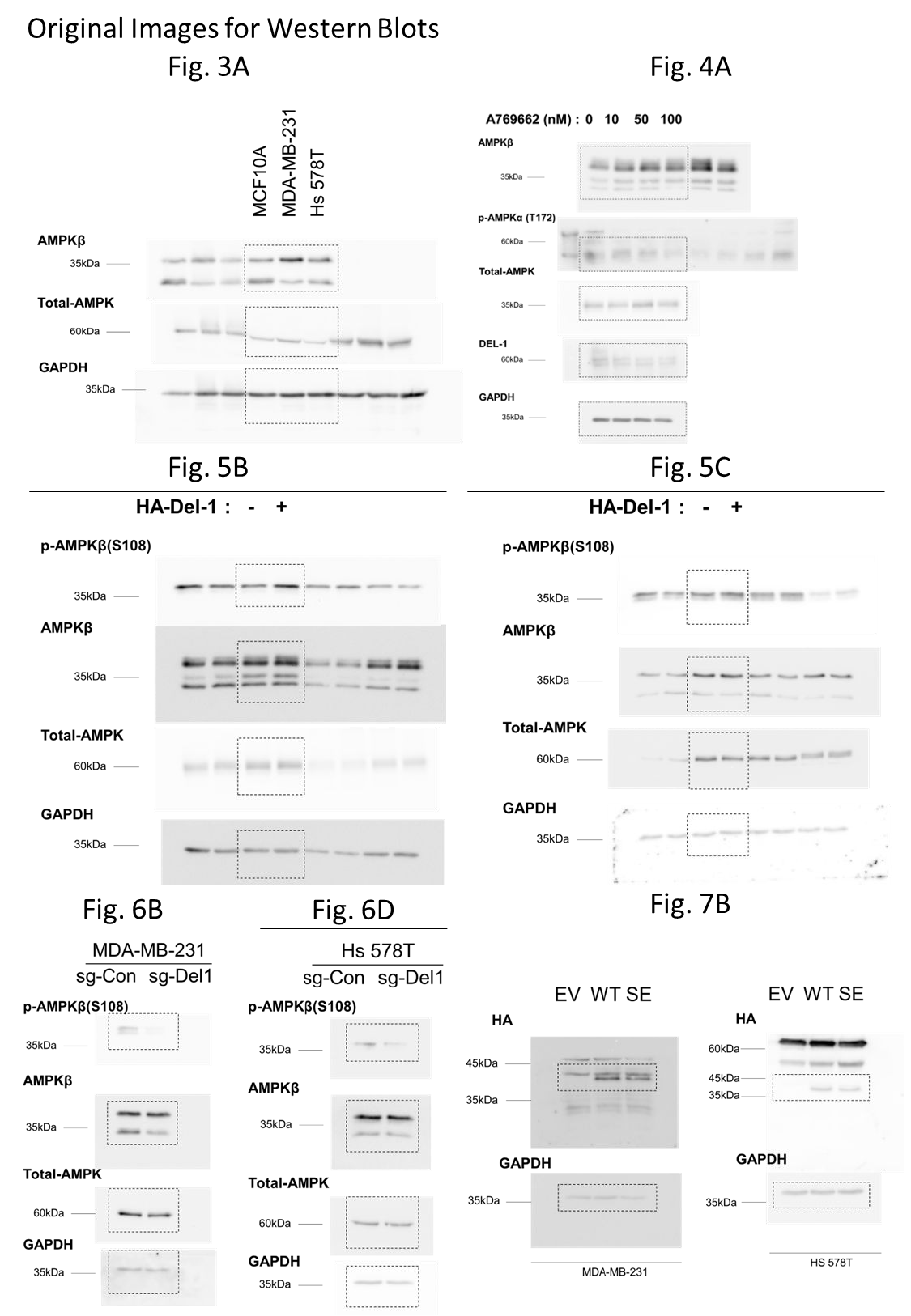

Supplement: Supplementary file 1 [file ijms-27-02679-s001.zip › ijms-4167764-supplementary.pptx]
